# Dynamic evolution and scenario simulation of habitat quality under the impact of land-use change in the Huaihe River Economic Belt, China

**DOI:** 10.1371/journal.pone.0249566

**Published:** 2021-04-05

**Authors:** Feng Tang, Meichen Fu, Li Wang, Wanjuan Song, Jiangfeng Yu, Yanbin Wu

**Affiliations:** 1 School of Land Science and Technology, China University of Geosciences, Beijing, China; 2 State Key Laboratory of Remote Sensing Science, Aerospace Information Research Institute, Chinese Academy of Sciences and Beijing Normal University, Beijing, China; 3 State Key Laboratory for Crop Genetics and Germplasm Enhancement, Jiangsu Plant Gene Engineering Research Center, Nanjing Agricultural University, Nanjing, Jiangsu, China; 4 School of Management Science and Engineering, Hebei University of Economics and Business, Shijiazhuang, Hebei, China; 5 GIS Big Data Platform for Socio-Economy in Hebei, Hebei University of Economics and Business, Shijiazhuang, Hebei, China; Institute for Advanced Sustainability Studies, GERMANY

## Abstract

Habitat quality is an important indicator for evaluating the biodiversity provided by ecosystem. Estimating and scenario-simulating the dynamic evolution and future development trends of habitat quality under the influence of land-use change is significant in regional biodiversity conservation, formulating land-use planning, and maintaining the ecological environmental sustainability. In this article, we included the Huaihe River Economic Belt as the area of study because of its vital location in China and applied the CA–Markov and InVEST models to analyze the spatio-temporal evolution of habitat quality and to simulate the future development trends of habitat quality under three different land-use scenarios: fast urban growth scenario, farmland conservation-oriented scenario, and ecological conservation-oriented scenario. The results showed that the land-use change in the Huaihe River Economic Belt was mostly represented by the continuous increase of the built-up area, whereas other land types all declined in area from 1995 to 2015. The land-use changes under these three abovementioned alternative future scenarios with different development orientations were considerably different. The built-up area has been shown to expand rapidly to occupy other land types on a large scale under the fast urban growth scenario. Urban land increased slightly and a large area of rural residential land would be converted into farmland under the farmland conservation-oriented scenario. The built-up area and farmland might decrease while woodland, grassland and water would increase in extent of areas under the ecological conservation-oriented scenario. Habitat quality has been shown to be generally poor, continuing to decline from 1995 to 2015, while its spatial distribution was higher in the southwest and northeast areas and lower in the central regions. The future habitat quality would display a downward trend under the fast urban growth and farmland conservation-oriented scenarios with a further deterioration of the ecological environment, while the ecological conservation-oriented scenario predicted the converse trend that the ecological environment would be improved productively. This study may be useful for understanding the impact of land-use dynamics on biodiversity. The research results can provide a scientific basis for the decision-makers to formulate biodiversity conservation and land management policies.

## Introduction

As a proxy for biodiversity, habitat quality refers to the ability of ecosystems to provide conditions appropriate for individual and population persistence, which mainly describes the survival environment of animal species rather than human populations [1]. It also determines the state of biodiversity, to a certain extent, and is an important indication of ecosystem services and health [2, 3]. As an important part of global change, land-use change has a significant impact on the quality of regional ecological environments and biodiversity conservation [4–6]. Land-use changes can affect the circulation of material and energy flows between habitat patches by modifying the structure and composition of regional habitats, thereby changing the production and service capabilities of these habitats, resulting in biodiversity decline [3, 7, 8]. Previous studies have shown that the habitat quality decreases with an increase in human-interfered land uses [9–11]. Therefore, assessing, monitoring, and scenario-simulating the spatio-temporal evolution of habitat quality in response to land-use change and future development trends has become an important point of reference for biodiversity conservation and land-use management.

Previous research programs focused on the evaluation of wildlife habitat quality and the impact of human activities, mainly through field investigations of biodiversity and the habitats themselves [12–14]. With international projects such as the International Geosphere-Biosphere Programme (IGBP), the International Human Dimensions Programme on Global Environmental Change (IHDP), and the World Climate Research Programme (WCRP), which takes Land Use/Land Cover Change (LULCC) as a core research issue to investigate global change, scholars have started to pay close attention to the impact of land-use change on regional habitat quality. The current research strategies for examining habitat quality can be categorized into two main categories. The first strategy was the comparative evaluation based on several computer models, such as ecological niche model, ecological suitability model, SolVES model and InVEST model [15–18]. The other strategy was to adopt a comprehensive index evaluation based method [19, 20]. Overall, the previous habitat quality research programs were based on survey data relating to wild animals and plants at a single point of time, establishing a habitat quality evaluation index system and scoring criteria (including species richness and vegetation coverage) to measure the habitat quality index, and conducting static studies on the quality level of regional or community habitats [21–23]. Because of the limitations in data accumulation and the research methods, less attention has been paid to the dynamic monitoring of habitat quality and its spatial changes [24]. In recent years, with the application of mathematical models and the advancements in Global Positioning System (GPS), Geographic Information System (GIS), and Remote Sensing (RS) technologies, scholars have started to pay attention to the spatio-temporal differences in habitat quality, particularly with the invention and widespread application of the InVEST model, which has promoted the development of habitat quality research [25, 26].

In terms of land-use, the existing models for predicting land-use change include system dynamics, artificial neural networks, GIS-optimization modeling, random prediction, empirical regression, Cellular Automata (CA)-Markov model, and conversion of land use and its effects (CLUE)-S. The CA-Markov model combines the ability of long-term predictions offered by the Markov model with the advantage of spatial change simulation of complex systems offered by the CA model. It can simulate land-use changes from different spatio-temporal scales and improve the prediction accuracy [27]. Therefore, the CA–Markov model has been widely applied in case studies [6, 28–30].

Current research on the responses to land-use change and habitat quality mainly focuses on analyzing the characteristics of its historical and present evolution, and there is insufficient research on long-term monitoring, future scenario simulation and the prediction of development trends in habitat quality. However, only a few scholars have carried out research on the prediction of habitat quality, mainly from the perspective of a single land-use scenario, without considering the possibility of different scenarios and the diversity of future regional development plans. The InVEST model and the CA–Markov model have achieved good evaluation results in their respective research fields, but the two have rarely been applied in combination in a study of the scenario simulation of regional habitat quality. In order to supplement these gaps in the literature, we attempted to combine the InVEST model and the CA-Markov model to carry out the study of habitat quality change under different development scenarios in the future, and selected a typical area to verify the effectiveness of this combination in the multi-scenario prediction of habitat quality.

The Huaihe River Basin is located between the Yangtze River Basin and the Yellow River Basin. It is an important grain production base and energy, mineral, and manufacturing base in China. The river basin is densely populated with 600 people/km^2^, which is far greater than the national population density of 148 people/km^2^, but the overall economic development is relatively slow [31]. For a long time, under the development model of economic priority, the non-point source pollution problem of industry and agriculture in the Huaihe River Basin was prominent, resulting in serious ecological damage and low environmental quality. Although China has started to increase its efforts towards developing ecological awareness in the recent years, the current state of ecological deterioration is difficult to reverse in the short term. In October 2018, the Chinese government released the "Huaihe River Economic Belt Development Plan" to promote the coordinated development of ecological conservation and the economy of the Huaihe River Basin, and is committed to building China’s fourth growth pole [32]. As a new field of research, the Huaihe River Economic Belt has entered scholarly focus, and various studies on this field need to be undertaken urgently.

Huaihe River Economic Belt plays an important role in China’s future development layout. However, there are few studies on this area, which leads to the lack of necessary scientific information and reference basis for government departments in this area to formulate future sustainable development strategies and management policies. Reasonable land-use policy is the basis of regional sustainable development, and habitat quality is an important indicator to measure regional ecological environment health. Therefore, grasping the evolution law of land use and habitat quality is of great value for local decision-makers to improve the sustainability of land resource utlization, biodiversity conservation and ecosystem management.

Given the above considerations, our primary goal was to provide scientific information and reference for local decision-makers of the Huaihe River Economic Belt to formulate rational land-use policies and biodiversity conservation strategies, based on the analysis of historical evolution characteristics and future development trends of land use and habitat quality. Thus, we have integrated the CA-Markov and InVEST models to evaluate and scenario-simulate the dynamic evolution of habitat quality under the impact of land-use changes. Our detailed research objectives were as follows: (1) to reveal the characteristics of past and present land-use change and simulate different land-use scenarios for the future, (2) to evaluate the historical and current spatio-temporal evolution of habitat quality and predict its future development trends under alternative scenarios, (3) to analyze the impact of land-use change on habitat quality in different scenarios, and (4) to put forward land management strategies and policy suggestions for the future development of the Huaihe River Economic belt.

## Materials and methods

### Study area

**Fig 1:**
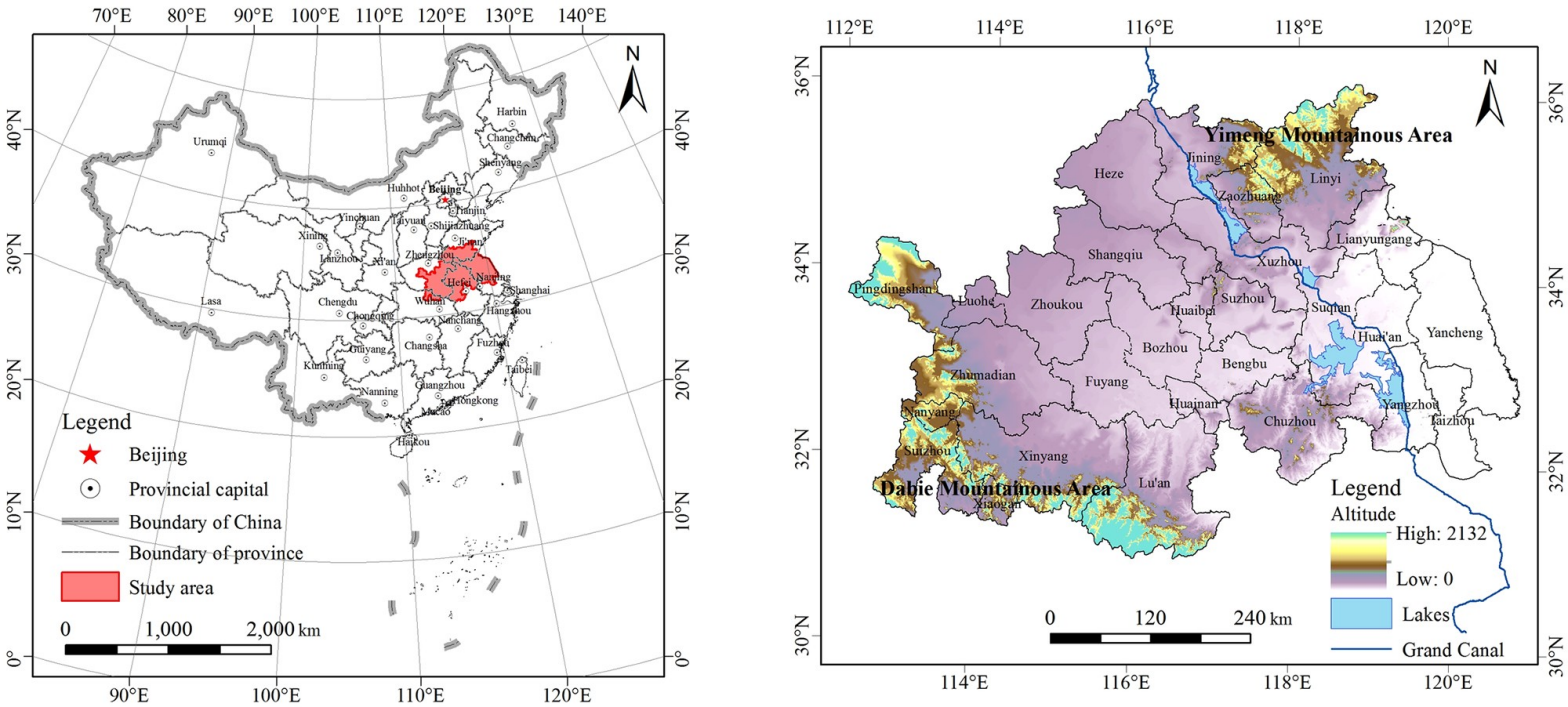
Location and elevation of the Huaihe River Economic Belt.

The Huaihe River Economic Belt (31°01′ to 36°12′N, 112°15′ to 120°54′E) covers the area where the main stream and first-class tributaries of Huaihe River and the Yishusi River Basin flow as the planning scope (Fig 1). It extends across 152 counties belonging to 28 prefecture-level cities in the five provinces of Jiangsu, Shandong, Anhui, Henan and Hubei, covering a total area of 243,000 km^2^. This area is located in the climate transition zone between the north and the south of China, and is rich in biodiversity [32]. The topography is higher in the west and lower in the east, and the terrain fluctuations are relatively minimal with vast plains. There are two large mountainous areas in the study area: one is the Dabie mountainous area located in the southwest, and the other is the Yimeng mountainous area located in the northeast. It has four large freshwater lakes, namely Nansi Lake, Luoma Lake, Hongze Lake, and Gaoyou Lake, along the Beijing-Hangzhou Grand Canal from the north to the south. At the end of 2017, the resident population was 146 million, accounting for 10.5% of China’s total population, and the per capita GDP was USD 6,921, sitting lower than the national average of USD 8,931. The Huaihe River Economic Belt is located in the core area of the Huaihe River Basin. It has many rivers and lakes and is rich in surface water resources, and there are nine national nature reserves in the study area. The local ecosystems and habitats are facing severe pressure and threats.

### Data sources and preprocessing

Four data sources were used in this study:

1. Land-use 1km×1km raster data in 1995, 2005 and 2015, and GDP and population density spatial distribution 1km×1km raster data in 2015, all provided by the Chinese Academy of Sciences’ Resource and Environmental Sciences Data Center (http://www.resdc.cn/). The land-use raster data are the classified remote sensing data product. They were generated by the Chinese Academy of Sciences’ Resource and Environmental Sciences Data Center based on Landsat remote-sensing images, using an artificial visual interpretation method. This data center divided land-use types into twenty-five classes, and provides free access to the public. The GDP spatial distribution raster data were determined by this data center on the basis of a comprehensive consideration of multiple factors such as land-use type, night light brightness, and residential density that are closely related to human economic activities, using the multi-factor weight method to distribute the county-level GDP statistics data into grid units [33]. The population density spatial distribution raster data were determined by this data center on the basis of a comprehensive consideration of multiple factors such as land-use type, night light brightness, and residential density that are closely related to population, using the multi-factor weight method to distribute the county-level demographic statistics data into grid units [34].
2. Annual average temperature and precipitation spatial distribution 1km×1km raster data, provided by the China Meteorological Science Data Center (http://data.cma.cn/).
3. Elevation, slope and aspect data extracted from the SRTM DEM 90m raster data downloaded from the China Geospatial Data Cloud Platform (http://www.gscloud.cn/).
4. Vector data including railways, highways, national roads, provincial roads and county roads, provided by the National Fundamental Geographic Information System (http://nfgis.nsdi.gov.cn).

With reference to the Land-Use/Land-Cover Remote Sensing Monitoring Data Classification System of the Chinese Academy of Sciences, and according to the research needs and actual land-use situation in the Huaihe River Economic Belt, we used the ArcGIS 10.2 platform (ESRI, Redlands, CA, USA) to reclassify the original raster data containing twenty-five land-use types into eight categories: farmland, woodland, grassland, water, urban land, rural residential land, industrial and traffic land, and barren land. In order to express them more clearly, we have further converted the urban land, rural residential land and industrial and traffic land into built-up area. The evaluation unit was a 1km×1km grid unit and all spatial data were processed as a 1km×1km raster layer on the ArcGIS 10.2 platform (ESRI, Redlands, CA, USA).

### Research methods

This study mainly started from the perspective of land-use change, focused on simulating the evolution trend of habitat quality in the Huaihe River Economic Belt under different development scenarios in the future. Therefore, there were two core research contents: one was the multi-scenario simulation of land-use change, and the other was the assessment of the habitat quality in a long-term series. Considering the advantages of the CA-Markov model in land-use prediction and the widespread recognition of the InVEST model in the evaluation of habitat quality, we used the CA-Markov model to simulate future land-use changes in the Huaihe River Economic Belt under three different development scenarios including fast urban growth scenario, farmland conservation-oriented scenario and ecological conservation-oriented scenario. We used the InVEST model to evaluate the habitat quality from 1995 to 2015 in the Huaihe River Economic Belt, and further analyzed the future change characteristics in habitat quality under the above mentioned three development scenarios.

#### Cellular Automata (CA)–Markov model

The Markov model is a traditional method for simulating land-use change. When we assume no aftereffects, the status of the land-use type in the study area at time t+1 depends only on the status of the land-use type at time t [28]. The land-use change process can be expressed as follows:

$$S_{\left(t+1\right)}=P\times S_{\left(t\right)}$$ (1)

where *S*_(*t*)_ and *S*_(*t*+1)_ are the column vectors of the land-use type status at time *t* and *t*+1, respectively, *P* is the land-use transition probability matrix, and its element *p*_*ij*_ is the transition probability of occurrence from type *i* to type *j*. Furthermore, *p*_*ij*_ can be calculated as follows:

$$P_{ij}=(A_{ij}/\sum_{j=1}^{n}A_{ij})\times 100\%$$ (2)

where *A*_*ij*_ is the area where type *i* converts to type *j* in the process of land-use change.

CA is a complex dynamic model that is discrete in time, space, and state, and has been widely used in land-use change simulation research. Its basic components include cell space, cell unit size, state set, state transition rule, and neighborhood range [27]. It can be expressed as follows:

$$S_{\left(t+1\right)}=f(S_{\left(t\right)},N)$$ (3)

where *S* is the set of finite and discrete cellular states, *S*_(*t*)_ and *S*_(*t*+1)_ are the system state results at time *t* and *t*+1, *N* is the cellular neighborhood range, and *f* is the state transition rule function of cellular interaction in the neighborhood range.

The CA–Markov model combines the respective advantages of the Markov model and the CA model [28]. Its working principle is to take the land use in the base period as the initial state based on the land-use transfer area in its previous stage and the land suitability atlas under the influence of multiple factors in the starting year [35]. The land-use type is then redistributed until the area predicted by the Markov chain is reached. This method solves the bottleneck problem of land-use spatio-temporal synchronous simulation. We used the CA–Markov module in the IDRISI Selva 17.0 software [36] to predict the land-use spatio-temporal pattern in the Huaihe River Economic Belt for different development scenarios in 2025. The simulation process had the following key steps:

1. Conversion rules: We used the land-use spatial data of 1995, 2005, and 2015 to calculate the land-use transfer area matrix and the transfer probability matrix for 1995 to 2005 and 2005 to 2015, which we then used as the conversion rules for predicting the land-use spatial pattern in 2015 and 2025.
2. Selection of driving factors for land-use change: Drawing on previous research results [18, 27], we selected 15 factors as the driving factors for land-use change from four aspects, namely topographic factors including elevation, slope, and aspect; meteorological factors including temperature and precipitation; distance factors including distance to water, distance to urban land, distance to rural residential land, distance to railway, distance to highway, distance to the national road, and distance to provincial roads and county roads; and socioeconomic factors including GDP and population density. The data of each driving factor were spatialized to prepare for the construction of the land-use suitability atlas.
3. Production of land-use suitability atlas: A logistic regression model is a nonlinear model often used in regression analysis of two-category or multicategory dependent variables and is currently widely used in the study of the forces driving land use and landscape change [17, 18, 24]. The regression equation is as follows:
  $$\text{logit}[P_{i}/\left(1-P_{i}\right)]=\beta_{0}+\beta_{1}x_{1}+\beta_{2}x_{2}+\cdot \cdot \cdot +\beta_{n}x_{n}$$ (4)
  where *P*_*i*_ is the probability of occurrence for land-use type *i* in each grid; *x*_1_, *x*_2_, …, *x*_n_ are driving factors that affect the land-use type transition; *β*_*0*_ is the constant; and *β*_1_, *β*_2_, …, *β*_*n*_ are the regression coefficients, which indicate driving direction and magnitude of the factors on land-use change in the grid unit. The regression analysis method calculates the probability of each land-use type in each grid, screens out the factors that have an important influence on the land-use pattern, and fits them to obtain the regression equation.
  Taking each land-use type as a dependent variable and the 15 driving factors as the independent variables, we used the LogisticReg module in IRISI32 to perform a logistic regression analysis to calculate the spatial distribution probability map of each land-use type, and then used the Collection Editor module in IRISI32 to integrate all the probability maps into a file to obtain the suitability atlas.
4. Construction of CA filter: Based on the relevant studies [28, 29, 37], we used a 5 × 5 filter.
5. Determination of the starting time and the number of CA cycles: We took the land-use patterns in 2005 and 2015, respectively, as the starting years to simulate the land-use pattern for 2015 and 2025, and in both cases, we set the number of CA cycles to 10.
6. Land-use simulation accuracy test: To ensure the reliability of the simulation results, the Kappa coefficient [27, 37] was used to test the precision of the simulated land-use pattern. The formula is expressed as follows:
  $$K\text{appa}=\lfloor \left(P_{o}-P_{c}\right)/\left(P_{p}-P_{c}\right)\rfloor$$ (5)
  where *P*_*o*_ is the correct ratio of correctly simulated grid units, *P*_*c*_ is the ratio of correctly simulated grid units under random conditions, and *P*_*p*_ is the ratio of correctly simulated grid units under ideal conditions, which is 100%. It is generally believed that a Kappa greater than 0.8 indicates strong consistency and an excellent simulation effect, and that a Kappa less than 0.4 indicates a poor simulation effect.
  We used the CrossTab module in IDRISI Selva 17.0 [36] to calculate the Kappa coefficient in 2015. We input the actual land-use map in 2015 and the land-use map in 2015 simulated by CA-Markov model into the CrossTab module. After that operation, the Kappa coefficient was 0.93, indicating that the simulation effect of the CA-Markov model in this study was excellent. Therefore, we used the validated CA-Markov model to predict land-use types in 2025.
7. Land-use scenario settings: We set up three different scenarios to predict the land-use spatio-temporal pattern in 2025. The first was the fast urban growth scenario. Under this scenario, the urban land expansion rate was 5.68%, which was consistent with the average annual growth rate of urban land in the Huaihe River Economic Belt during the rapid urbanization period from 2005 to 2015 [24, 31]; during this period, farmlands and ecological lands (defined as woodland, grassland, and water) were in rapid decline and were being converted into urban lands. The second scenario was the farmland conservation-oriented scenario. In this instance, farmlands would have been effectively protected and increased due to the strict implementation of the farmland occupation and compensation balance policy, and the urban lands expansion rate would be 1.45%, which was consistent with the average annual growth rate of the urban population in the Huaihe River Economic Belt from 2005 to 2015 [24, 31]. The third scenario was the ecological conservation-oriented scenario, within which the ecological lands would have been effectively protected, urban lands would no longer grow in areas, rural residential lands and industrial and traffic lands would be further reduced, while areas covering woodlands, grasslands, and water would increase.

#### InVEST model

The InVEST model, which is used to evaluate ecosystem function [26], was jointly developed by Stanford University, The Nature Conservancy, and the World Wildlife Fund. The habitat quality module in the InVEST model calculates the habitat quality index by combining landscape type sensitivity and external threat intensity, and evaluates biodiversity service functions based on the index: the greater the index, the higher the habitat quality and the greater the biodiversity [26, 38]. The equation is as follows:

$$Q_{xj}=H_{j}\lfloor k^{Z}/\left(D_{xj}^{Z}+k^{Z}\right)\rfloor$$ (6)

where *Q*_*xj*_ is the habitat quality of grid *x* in land-use type *j*, *D*_*xj*_ is the threat level of grid *x* in land-use type *j*, *H*_*j*_ is the habitat suitability of land-use type *j*, *k* is a half-saturation constant that is usually half of the maximum value of *D*_*xj*_, and *z* is the normalisation constant, which is usually 2.5. *D*_*xj*_ can be calculated as follows:

$$D_{xj}=\sum_{r=1}^{R}\sum_{y=1}^{Y_{r}}\left(w_{r}/\sum_{r=1}^{R}w_{r}\right)r_{y}i_{rxy}\beta_{x}S_{jr}$$ (7)

where *R* is the number of threat factors, *y* is the number of grids on the raster layer of the threat factor *r*, *Y*_*r*_ is the number of grids occupied by the threat factor on the land-use type layer, *w*_*r*_ is the weight of the threat factor, *r*_*y*_ is the threat factor value of grid *y*, *i*_*rxy*_ is the habitat threat level of grid *x* from threat factor *r* on grid *y*, *β*_*x*_ is the reachability level of grid *x*, and *S*_*jr*_ is the sensitivity of land-use type *j* to threat factor *r*. Here, *i*_*rxy*_ can be calculated as follows:

$$i_{rxy}=1-(d_{xy}/d_{r\text{max}})\;if\;linear$$ (8)

$$i_{rxy}=1-(d_{xy}/d_{r\text{max}})\;if\;exponential$$ (9)

where *d*_*xy*_ is the straight line distance between grid *x* and grid *y* and *d*_*rmax*_ is the maximum influence distance of threat factor *r*.

The first key issue to run the InVEST habitat quality module is to determine the threat factors. In this study, we mainly analyzed habitat quality changes from the perspective of land-use change. The Huaihe River Economic Belt is an important agricultural production base and a cluster of industrial cities in China. In the context of rapid urbanization and industrialization, the expansion of construction land and the development of industrial and agricultural activities are the main sources of habitat threat. Therefore, we selected farmlands, urban lands, rural residential lands, and industrial and traffic lands as the threat factors. The second key issue was the consideration of model parameters. Based on the relevant literature [3, 11, 24, 27, 38–42] and the expert knowledge, we assigned the maximum stress distance and weight of each threat factor, and assigned the sensitivity of various habitat types to the threat factors (Tables 1 and 2). To facilitate the comparisons of habitat quality changes, and using the equal interval method, we divided the habitat quality index into five grades: V (0 to 0.2), IV (0.2 to 0.4), III (0.4 to 0.6), II (0.6 to 0.8), and I (0.8 to 1). From grade I to grade V, the habitat quality worsened.

**Table 1:** Maximum distance, weight and spatial decay type of the threat factors affecting habitat quality.

Threat factor	Maximum distance (km)	Weight	Spatial decay type
Farmland	6	0.6	Exponential
Urban land	10	0.9	Exponential
Rural residential land	8	0.7	Exponential
Industrial and traffic land	12	1	Linear

**Table 2:** The habitat suitability and sensitivity of land-use type to each threat factor.

Land-use type	Habitat suitability	Farmland	Urban land	Rural residential land	Industrial and traffic land
Farmland	0.3	0	0.6	0.4	0.5
Woodland	1	0.7	0.9	0.8	0.9
Grassland	0.6	0.4	0.5	0.4	0.5
Water	0.8	0.7	0.9	0.8	0.9
Urban land	0	0	0	0	0
Rural residential land	0	0	0	0	0
Industrial and traffic land	0	0	0	0	0
Barren land	0.2	0.2	0.5	0.4	0.3

## Results

### Land-use change in the Huaihe River Economic Belt

#### Spatio-temporal characteristics of land-use change from 1995 to 2015

**Fig 2:**
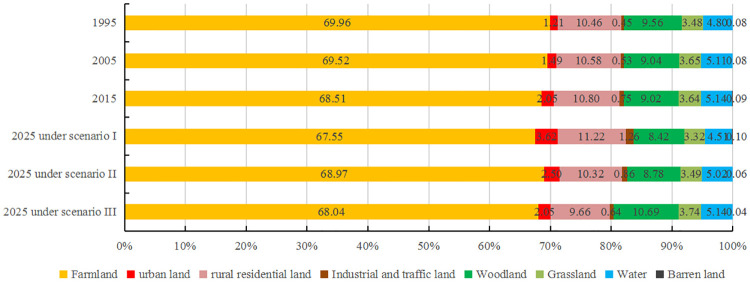
Area ratio of each land-use type in 1995, 2005, 2015 and 2025 (Note: Scenario I represents fast urban growth scenario, scenario II represents farmland conservation-oriented scenario, scenario III represents ecological conservation-oriented scenario).

Land use was dominated by farmlands and built-up areas, accounting for approximately 82% of the total area, and barren lands were rarely less than 1% (Fig 2). From the perspective of spatial distribution, farmlands comprised about 68% of the total area and were distributed almost everywhere in the study area. Rural residential lands were around 11% of the total area and were scattered throughout the study area in the form of dots. Urban lands were less than 3% but showed a rapid growing trend. Woodlands and grasslands were mainly distributed in the southwest and northeast mountainous regions, and water was mainly distributed in the middle and lower reaches of the Huaihe River and the lake area along the Beijing-Hangzhou Grand Canal. From 1995 to 2015, the extent of area, where land-use changes occurred was about 4195 km^2^ in the Huaihe River Economic Belt. Land-use changes were mostly represented by the rapid increase of built-up areas, concomitant with the continuous decrease of farmlands, woodlands and grasslands. Changes in water and barren lands were relatively stable.

#### Land-use change under different future scenarios

**Fig 3:**
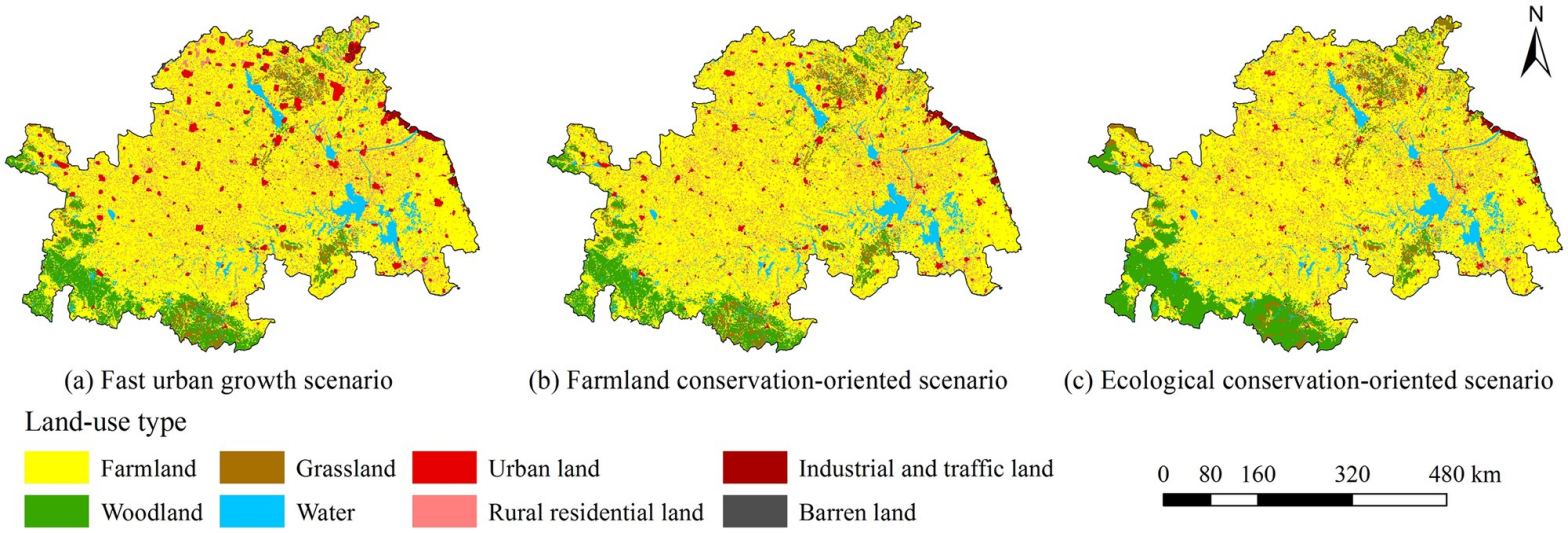
Spatial distribution of land use in 2025 in (a) fast urban growth scenario; (b) farmland conservation-oriented scenario; and, (c) ecological conservation-oriented scenario.

Land-use change in the Huaihe River Economic Belt under different future scenarios simulated by the CA–Markov model was considerably different (Fig 3). Under the fast urban growth scenario (Fig 3a), urban lands expanded rapidly outwards and occupied other land cover types. From 2015 to 2025, urban lands would be increased by 3815 km^2^, rural residential lands would be increased by 1020 km^2^, industrial and traffic lands would be increased by 1239 km^2^. The other land types all decreased in area compared to 2015, with farmland decreasing the most by 2333km^2^, and woodland, grassland and water decreasing by 1458km^2^, 777km^2^, and 1530km^2^, respectively. Under the farmland conservation-oriented scenario (Fig 3b), urban land expansion would be increased by only 1093 km^2^, while industrial and traffic lands would be increased by 267 km^2^, and rural residential lands would be decreased by 1166 km^2^. Farmlands were successfully protected and showed a slight increase of 1118 km^2^, which was mainly attributed to the consolidation of abandoned rural residential lands. Woodlands, grasslands and areas with water decreased by 2.66%, 4.12% and 2.33%, respectively. Under the ecological conservation-oriented scenario (Fig 3c), urban lands remained almost unchanged, while rural residential lands, industrial and traffic lands and farmlands were reduced by 10.56%, 14.67% and 0.69%, respectively, and were mainly converted to ecological lands. Woodlands would be increased the most by 4058 km^2^, while grasslands and areas with water would be increased slightly, and 122 km^2^ of barren lands would be converted as woodlands.

### Habitat quality change in the Huaihe River Economic Belt

#### Spatio-temporal change of habitat quality from 1995 to 2015

The overall habitat quality in the study area was poor and dominated by grade IV and grade V. The habitat quality average values in 1995, 2005, and 2015 were 0.3662, 0.3618, and 0.3538, respectively, indicating that the habitat quality in the study area has been continuously declining.

**Fig 4:**
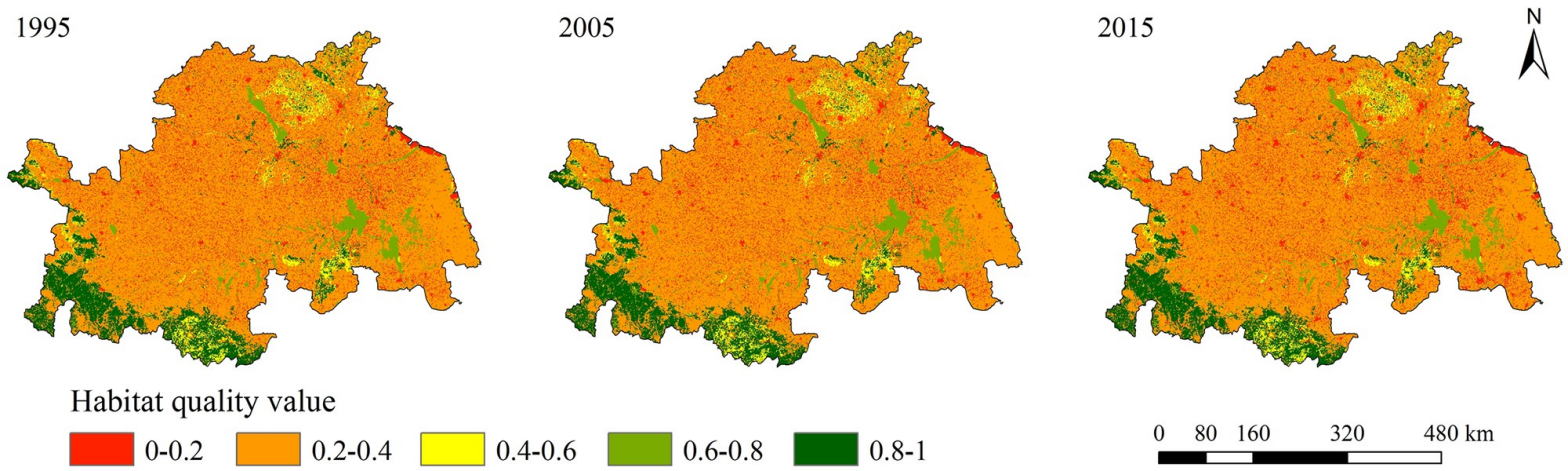
Spatial distribution of habitat quality in 1995, 2005 and 2015.

**Fig 5:**
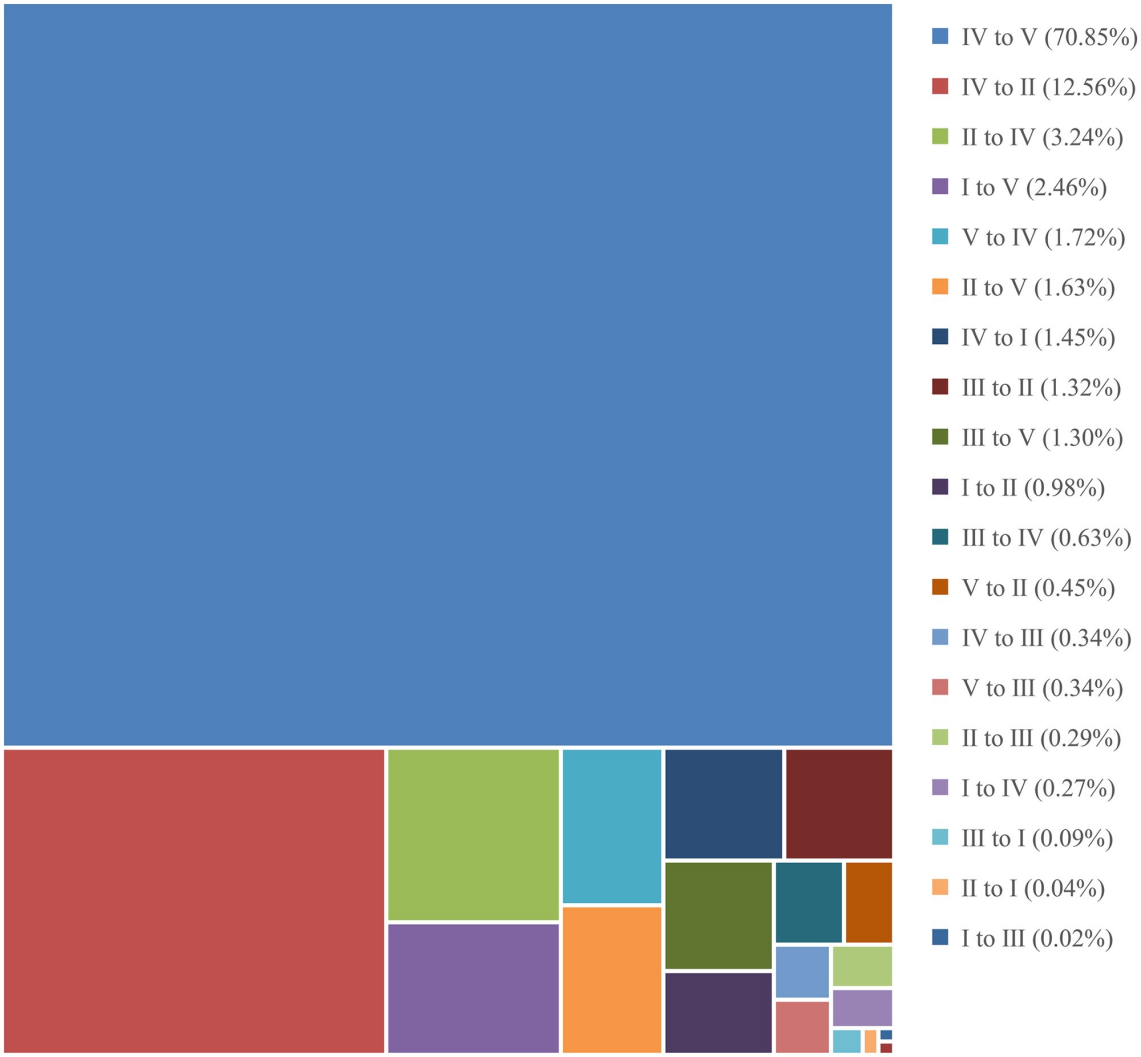
Area ratio of different change directions of habitat quality grades from 1995 to 2015.

As per the spatial distribution patterns (Fig 4), the habitat quality was higher in the southwest and northeast areas but lower in the middle regions. The habitat quality distribution was highly consistent with the distribution characteristics of land-use types. Grade I and grade II were concentrated in the Dabie mountainous area in the southwest, the middle and lower reaches of the Huaihe River, and the lake distribution area including Nansi Lake, Hongze Lake, Gaoyou Lake, and Luoma Lake along the Beijing-Hangzhou Grand Canal. These areas were marked by a concentrated distribution of woodland and water, which were less affected by human interference and generally had higher habitat quality. Grade III was mainly distributed in the Yimeng mountainous area in the northeast, and some were distributed in the Dabie mountainous area, and the land types in these regions were dominated by grasslands. Grade IV was distributed almost everywhere in the study area and the land types were dominated by farmlands. Grade V was scattered throughout the study area in the form of clumps and dots, and the spatial distribution coincided strongly with the distribution of the built-up areas and barren lands. From 1995 to 2015, the extent of area where changes in habitat quality occurred was about 4190 km^2^ in the Huaihe River Economic Belt. The changed areas were not concentrated, but scattered throughout the study area in the form of dots. The habitat quality changes during this period have mostly been represented by the increase in grade V, and the varying degrees of reduction of the other four grades (Fig 5). The most prominent direction of change was the conversion of grade IV to grade V, accounting for about 71% of the total changed areas, mainly because of the continuous shrinkage of habitat patches because of the large-scale expansion of built-up areas.

#### Habitat quality under different land-use scenarios

**Fig 6:**
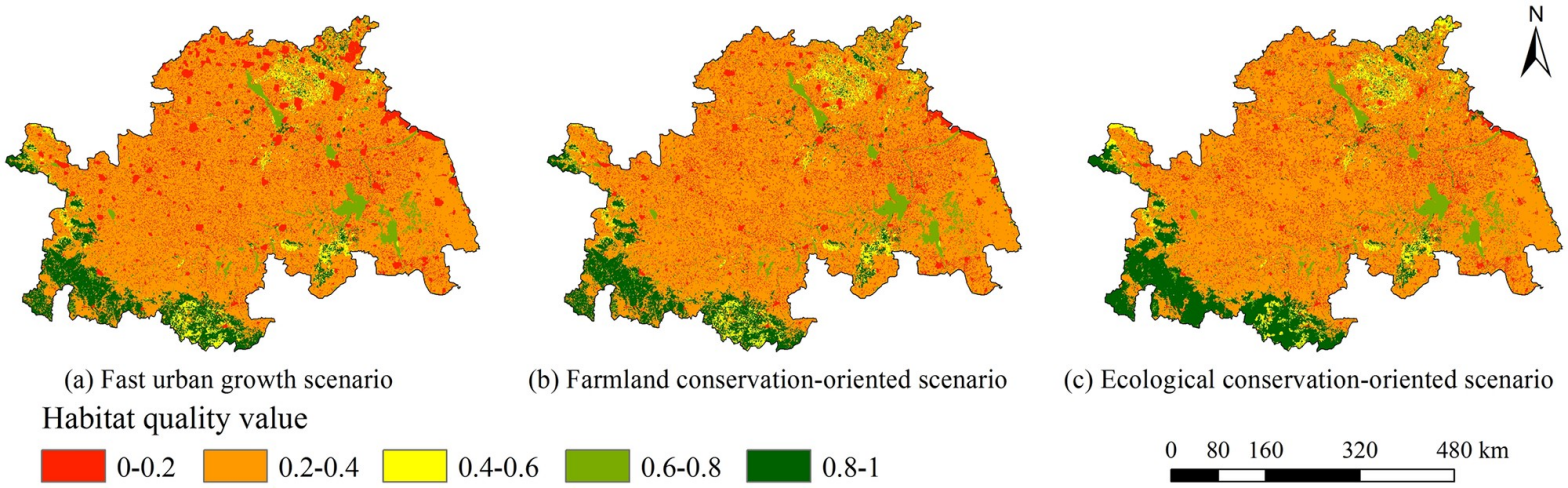
Spatial distribution of habitat quality in 2025 in (a) fast urban growth scenario, (b) farmland conservation-oriented scenario, and (c) ecological conservation-oriented scenario.

**Fig 7:**
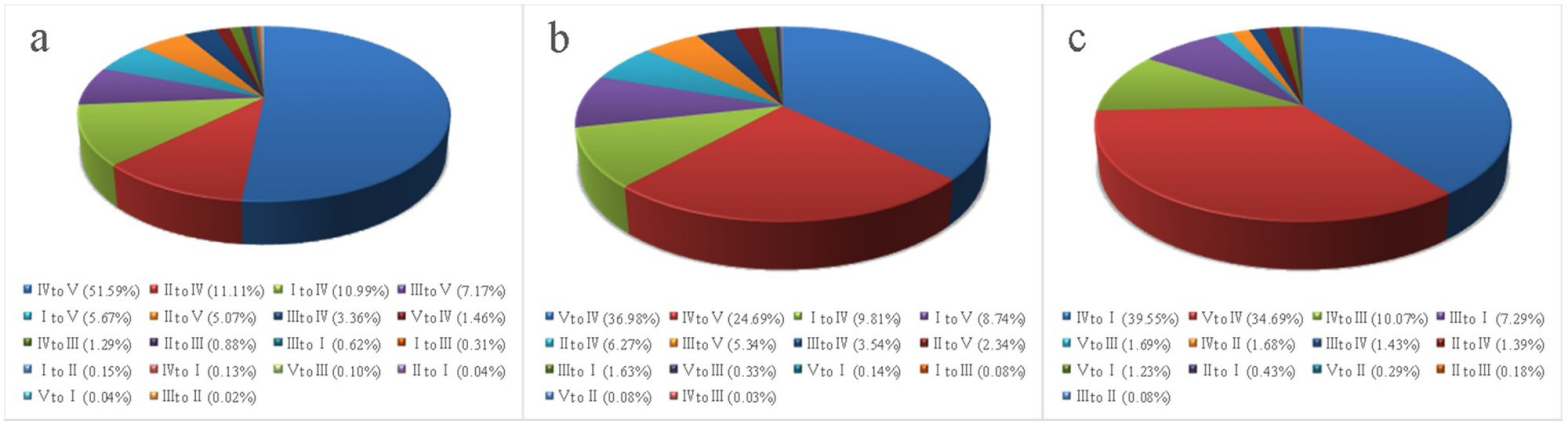
Area ratio of different change directions of habitat quality grades from 2015 to 2025 in (a) fast urban growth scenario, (b) farmland conservation-oriented scenario, and (c) ecological conservation-oriented scenario.

The future habitat quality changes under different land-use scenarios in the Huaihe River Economic Belt simulated by the combination of the CA–Markov and InVEST models were significantly different (Figs 6 and 7). Under the fast urban growth scenario, the average values of habitat quality in 2025 was 0.3403, decreasing by 0.0135 as compared to that in 2015. Under the farmland conservation-oriented scenario, the average values of habitat quality in 2025 was 0.3529, decreasing by 0.0009 as compared to that in 2015. Under the ecological conservation-oriented scenario, the average values of habitat quality in 2025 was 0.3715, increasing by 0.0177 as compared to that in 2015. The future habitat quality in the study area showed a downward trend under the fast urban growth scenario and farmland conservation-oriented scenario, and the ecological environment may further deteriorate. In contrast, it showed a growth trend under the ecological conservation-oriented scenario, with the ecological environment developing productively.

From 2015 to 2025, under the fast urban growth scenario (Fig 7a), the changes in habitat quality occurred in about 4651 km^2^ of the study area. The areas of change were mostly concentrated around the urban lands. The habitat quality change was represented by a significant increase in grade V areas, and varying degrees of reduction among the other four grades. The most prominent change direction was the conversion of grade IV to grade V, accounting for about 52% of the total change area, mainly because of the large-scale conversion of farmlands near cities to urban lands due to the rapid urbanization under this scenario. Under the farmland conservation-oriented scenario (Fig 7b), from 2015 to 2025, the changes in habitat quality occurred in about 3438 km^2^ of the study area, and the changed areas were scattered. The most prominent direction of the change was the conversion between grade IV and grade V, accounting for about 62% of the total change area, mainly because in this scenario, although the urbanization growth rate was slowed, some farmlands would be still occupied by urban expansion, and at the same time, a large quantity of rural residential lands would be converted into farmlands due to the need for farmland protection. Compared with the above mentioned two scenarios, the change areas of habitat quality under the ecological conservation-oriented scenario were larger, reaching 6345 km^2^ (Fig 7c). From 2015 to 2025, the habitat quality change was represented by a significant decrease of grade V and grade IV, and varying degrees of increase among the other three grades. Grade I would be increased by 4047 km^2^, accounting for about 64% of the total change area. This phenomenon was mainly attributed to the large quantity of farmlands, rural residential lands and industrial and traffic lands converted into woodlands due to the need for ecological protection.

## Discussion

### Land-use change affecting habitat quality

From 1995 to 2015, the habitat quality was changed in about 4190 km^2^ of the study area. At the same time, land-use was changed in about 4195 km^2^ of the study area. By spatially superimposing the habitat quality and the land-use change areas in the ArcGIS 10.2 platform (ESRI, Redlands, CA, USA), we found that the overlap rate of the two was 99.88%, indicating that land-use change was the main driver of the habitat quality change. Degradation was the main feature of habitat quality changes from 1995 to 2015 in the study area, which was closely related to the expansion of the built-up areas. The increase of habitat quality in a very few areas benefitted from the local growth of ecological lands such as woodlands, grasslands and areas with water. Sun et al. [11] used the InVEST model to monitor the spatio-temporal dynamics of habitat quality in the Nansihu Lake Basin and analyzed the impact factors of habitat quality change. Xu et al. [3] used the InVEST model to evaluate habitat quality and analyzed the impacts of land-use change on habitat quality during 1985 to 2015 in the Taihu Lake Basin. Yan et al. [18] used the logistic multiple regression model to analyze the driving forces of the changes in habitat quality in the northeast area of Beijing. Moreira et al. [38] used the InVEST model to assess the habitat conservation status in Pico Island from the perspective of land-use. Sallustio et al. [43] assessed habitat quality and degradation based on a high-resolution land-use map of Italy. Aneseyee et al. [44] used the InVEST model to evaluate the spatio-temporal evolution of habitat quality in the Omo-Gibe Basin of Ethiopia, and used the Pearson’s correlation coefficient to analyze the correlation of the habitat quality and the impact factors. All of them found that the land-use change was the main driving factor for regional habitat quality degradation, which was consistent with our results.

### Habitat quality future simulation

Habitat quality is an important indicator to measure the health of the regional ecological environment. Predicting the future trends of habitat quality change can provide a scientific basis for the government to formulate land-use policies that are conducive to maintaining biodiversity and preventing the deterioration of regional ecological integrity. Li et al. [25] combined the SLEUTH and InVEST models to simulate the future habitat quality of Changzhou City, mainly from the perspective of the urban growth scenario. This kind of study, using a single scenario simulation to obtain the degradation of future habitat quality, can also provide a reference for formulating regional land-use policies, but it lacks the diversity of options for decision-makers. With the increasing awareness of farmland protection and ecological protection in China [45, 46], the traditional urban development model through the long-term unrestricted and fast-growing built-up areas might change. Land-use change trends in the future may have more different possibilities. The characteristics of future habitat quality changes under different land-use scenarios are different. These issues have highlighted the limitations of single scenario research.

We used a combination of the CA–Markov and InVEST models to simulate the future change trends of habitat quality in the Huaihe River Economic Belt under three different land-use scenarios: the fast urban growth scenario, the farmland conservation-oriented scenario and the ecological conservation-oriented scenario. This approach would provide decision-makers with multiple options to adjust land-use policies to formulate future development plans. It also effectively makes up for the shortcomings of single land-use scenario simulations. Our study found that the development trends of future habitat quality under the different land-use scenarios in the Huaihe River Economic Belt would be very different. Under the fast urban growth scenario, the future habitat quality will drop sharply, whereas the biodiversity degradation pressure would be particularly higher in the surrounding areas of the urban lands and the ecological environment would deteriorate further. In the future, it will be necessary to focus on the monitoring of the dynamic changes of land use in the sub-urban lands under this scenario. Under the farmland conservation-oriented scenario, the expansion rate and area of future urban lands would be significantly lower than those in the fast urban growth scenario, and the farmlands would be effectively protected. The projected habitat quality in 2025 would be slightly decreased as compared to that of 2015 under this scenario, and the risks of biodiversity degradation in the future could be relatively lesser. Under the ecological conservation-oriented scenario, the future habitat quality would increase, where the biodiversity and ecological environment will be improved. The expansion of built-up areas would have been curbed under this scenario, and the further development of cities should abandon the traditional model of extensional expansion and embark on the path of intensively using land.

### Policy implications

The social and economic development of the Huaihe River Economic Belt is ever growing and land use is constantly changing, as a result, the decline of habitat quality in the study area is becoming increasingly prominent. To protect the biodiversity in the Huaihe River Economic Belt and achieve a sustainable development, it is suggested that the corresponding scientific land management strategies should be implemented in a targeted manner, according to the regional differences in habitat quality.

In the southwest and northeast mountainous regions, the areas along the Huaihe River and along the Beijing-Hangzhou Grand Canal, the land-use types are mainly woodlands and water, which are important habitat gathering areas with high habitat quality (grade I and grade II). For these areas, the occupation of ecological lands by built-up areas should be strictly controlled, and extremely important habitat patches and ecological spaces such as forests, lakes and rivers should be vigorously protected. In addition, for some habitat patches such as bottomland and shallows, which are small and easily occupied by urban development, attention needs to be paid to their important role in maintaining biodiversity and habitat diversity [3]. The protection of these above areas with high habitat quality and rich biodiversity can be strengthened through government intervention, such as the delineation of ecological protection zones by government departments to limit the interference of human activities in these areas [47]. In areas with grade IV of habitat quality, where farmland is the main land-use type. For these areas, although the habitat quality is poor, as the core carrier of agricultural production, farmland plays an important role in ensuring food security. Especially for China, a country with a large population, farmland must be effectively protected in order to achieve food security and maintain social stability. The Huai River basin is an important grain production base in China, the occupation of farmlands by built-up areas needs to be reduced in the future. It is recommended to stabilize the agricultural space and reduce the loss of important farmland resources by strictly controlling farmland transition and implementing the farmland occupation and compensation balance policy. In addition, the local government of the Huaihe River Economic Belt should delimit the high-quality farmland resources as the permanent basic farmland protection zones to strengthen the construction of farmland ecosystem [48]. In areas with grade V of habitat quality, where built-up area is the main land-use type. For these areas, it is necessary to optimize the allocation of land use on the premise of understanding the risk of biodiversity loss. Delineation of urban growth boundary is regarded as an effective way to control disorderly urban sprawl and curb biodiversity decline, our results can provide an important reference for the urban planning process of the Huaihe River Economic Belt. Specifically, local decision-makers and planners should select districts with low habitat quality as built-up areas, and the urban growth boundary can be determined on the basis of a given area of construction land requirements [3]. This study has predicted the demand quantity and distribution pattern of urban lands under three different land-use scenarios in the future, which provides decision-makers with a variety of options for formulating different urban growth boundaries.

There are 28 cities of the Huaihe River Economic Belt, and their development mode, socio-economic conditions, and biodiversity problems differ significantly. In the process of land-use policy formulation, it is necessary to highlight the characteristics of each city, the economic benefits and ecological benefits must be balanced. We have proposed three different land-use scenarios and development orientations, and predicted their differences in future land-use patterns and habitat quality changes. These cities can refer to our results to choose a suitable land-use development mode. Cities that have high economic level but low habitat quality, such as Yangzhou, Taizhou, Xuzhou, Yancheng, Huai’an and Zaozhuang, are suitable for choosing the ecological conservation-oriented development scenario. These cities should give priority to ecological benefits in the future development process, enhance habitat quality and strengthen biodiversity conservation by optimizing land-use structure and layout. The extensional expansion of built-up area is restricted in this development scenario, the utilization efficiency of existing built-up area must be improved to meet the land demand of economic growth and construction activities. Cities that have high habitat quality but low economic level, such as Lu’an, Xiaogan, Suizhou, Nanyang and Xinyang, are suitable for choosing the fast urban growth development scenario. These cities have a weak economic foundation but good ecological background conditions, in the short-term, the pursuit of rapid development is the main goal of these cities. In the process of formulating land-use policy, the requirements for ecological protection can be appropriately reduced so as not to hinder economic development in these economically backward cities. Although this development scenario allows the increase of built-up area, it is necessary to avoid blind and disorderly expansion of cities, prevent the destruction of important habitat patches, and use land planning to guide the rational use of construction land. Most of the cities located in the middle regions of the Huaihe River Economic Belt, such as Heze, Shangqiu, Zhoukou, Zhumadian, Fuyang, Bozhou, Huaibei, Suzhou, Bengbu and Suqian, are the gathering areas of farmland. Protecting farmland to ensure food security is an important task for these cities, they are suitable for choosing the farmland conservation-oriented development scenario.

### Limitations and outlook

The current study explored the characteristics of the spatio-temporal evolution of habitat quality and predicted its future development trends under different land-use change scenarios in the Huaihe River Economic Belt by using the CA–Markov and InVEST models. The combination of these models provided an effective method for monitoring and scenario-simulating the dynamic evolution of the regional land-use patterns and their respective habitat quality. Yet, this study still has some limitations. Regarding the technical management, there are strict artificial boundaries between different land-use types. However, these boundaries could not be incorporated very clearly under the natural conditions. This kind of fuzzy boundary zone is usually accompanied by more active material-flow and energy-flow exchange, and the fluctuation of biodiversity is also more intense. In the process of assessing the habitat quality, these areas were not differentiated, and this part of the research needs to be detailed in the future. In general, the InVEST model has become more mature with the widespread application, and it is superior to traditional methods used in spatial expression and dynamic research. However, the parameter setting in the calculation process has certain subjectivity, parameter verification and rationality evaluation are worth discussing in depth. In addition, the InVEST model estimates habitat quality by accumulating the effects of various threat factors, but the simple accumulation of the effects of each threat factor is not precisely equal to the comprehensive effect of each threat factor on the habitat quality. Under some conditions, the collective effect of multiple threats may be considerably greater than the sum of individual threats. The InVEST model does not consider this effect, and it needs to be improved for future applications. In order to reduce these limitations of the InVEST model for evaluating habitat quality, it is necessary to further supplement and optimize the model parameters by strengthening the collection of species occurrence data and considering the impact of specific human activities on habitat quality. Using the species distribution model to improve the precision of the InVEST model. That way, we could identify the habitats that target species inhabit more clearly, and conduct a more subtle evaluation for the effect of land-use change on these habitats. In addition, obtaining first-hand biodiversity and habitat information through field investigation is an important alternative strategy.

## Conclusions

This study integrated the CA–Markov and InVEST models to analyze the dynamic evolution characteristics of land-use and habitat quality from 1995 to 2015 in the Huaihe River Economic Belt, and further simulated their spatio-temporal patterns and development trends under three different land-use scenarios. On the basis of the above analysis, land management and biodiversity conservation strategies in line with the different development orientations of the study area were put forward. Several conclusions were made as follow. The land-use change during the past 20 years in the Huaihe River Economic Belt was dominated by the continuous increase in built-up areas and decrease in farmlands and ecological lands, simultaneously, the habitat quality showed a continuous decline, indicating the impact of land-use dynamics on the habitat quality change. Changes in land use and habitat quality under different land-use scenarios and development orientations projected for 2025 were significantly different. The future habitat quality showed a dramatic downward trend with the built-up areas would be expanded rapidly under the fast urban growth scenario, the future habitat quality showed a slight downward trend with the urban lands would be expanded slowly and farmlands would be successfully protected under the farmland conservation-oriented scenario, the future habitat quality showed an upward trend with the ecological lands would be increased and urban lands remained almost unchanged under the ecological conservation-oriented scenario. The findings from this study offered the potential to analyze the impact of land-use dynamics on biodiversity. The results have provided scientific information and basis for local government and decision-makers of the Huaihe River Economic Belt to optimizing regional ecological environment, formulating rational land-use policies and biodiversity conservation strategies.

## References

[1] Johnson MD. Measuring Habitat Quality: A Review. Condor. 2007; 109(3): 489–504. 10.1093/condor/109.3.489

[2] Polasky S, Nelson E, Pennington D, Johnson KA. The impact of land-use change on ecosystem services, biodiversity and returns to landowners: a case study in the state of minnesota. Environmental & Resource Economics. 2011; 48(2): 219–242. 10.1007/s10640-010-9407-0

[3] Xu LT, Chen SS, Xu Y, Li GY, Su WZ. Impacts of Land-Use Change on Habitat Quality during 1985–2015 in the Taihu Lake Basin. Sustainability. 2019; 11(13): 3513–3533. 10.3390/su11133513

[4] Cotter M, Häuser I, Harich FK, He P, Sauerborn J, Treydte AC, et al. Biodiversity and ecosystem servicess-A case study for the assessment of multiple species and functional diversity levels in a cultural landscape. Ecological Indicators. 2017; 75: 111–117. 10.1016/j.ecolind.2016.11.038

[5] Daily GC, Polasky S, Goldstein J, Kareiva PM, Mooney HA, Pejchar L, et al. Ecosystem services in decision making: Time to deliver. Frontiers in Ecology and the Environment. 2009; 7(1): 21–28. 10.1890/080025

[6] Gashaw T, Tulu T, Argaw M, Worqlul AW. Modeling the hydrological impacts of land use/land cover changes in the Andassa watershed, Blue Nile Basin, Ethiopia. Science of The Total Environment. 2018; 619–620: 1394–1408. 10.1016/j.scitotenv.2017.11.191 29734616

[7] Aguilar R, Cristobal-Perez EJ, Balvino-Olvera FJ, de Jesus Aguilar-Aguilar M, Aguirre-Acosta N, Ashworth L, et al. Habitat fragmentation reduces plant progeny quality: a global synthesis. Ecology Letters. 2019; 22(7): 1163–1173. 10.1111/ele.13272 31087604

[8] Liu Y, Huang XJ, Yang H, Zhong TY. Environmental effects of land-use/cover change caused by urbanization and policies in Southwest China Karst area-A case study of Guiyang. Habitat International. 2014; 44: 339–348. 10.1016/j.habitatint.2014.07.009

[9] Petrosillo I, Zaccarelli N, Semeraro T, Zurlini G. The effectiveness of different conservation policies on the security of natural capital. Landscape and Urban Planning. 2009; 89(1–2): 49–56. 10.1016/j.landurbplan.2008.10.003

[10] Romero-Calcerrada R, Luque S. Habitat quality assessment using Weights-of-Evidence based GIS modelling: The case of Picoides tridactylus as species indicator of the biodiversity value of the Finnish forest. Ecological Modelling. 2006; 196(1–2): 62–76. 10.1016/j.ecolmodel.2006.02.017

[11] Sun XY, Jiang Z, Liu F, Zhang DZ. Monitoring spatio-temporal dynamics of habitat quality in Nansihu Lake basin, eastern China, from 1980 to 2015. Ecological Indicators. 2019; 102: 716–723. 10.1016/j.ecolind.2019.03.041

[12] Engel DW, Thayer GW, Evans DW. Linkages between fishery habitat quality, stress, and fishery populations. Environmental Science & Policy. 1999; 2(6): 465–475. 10.1016/s1462-9011(99)00043-x

[13] Goertz JW. Influence of Habitat Quality upon Density of Cotton Rat Populations. Ecological Monographs. 1964; 34(4): 359–381. 10.2307/2937068

[14] Vellend M, Lilley PL, Starzomski BM. Using subsets of species in biodiversity surveys. Journal of Applied Ecology. 2007; 45(1): 161–169. 10.1111/j.1365-2664.2007.01413.x

[15] Adhikari D, Tiwary R, Singh PP, Upadhaya K, Singh B, Haridasan KE, et al. Ecological niche modeling as a cumulative environmental impact assessment tool for biodiversity assessment and conservation planning: A case study of critically endangered plant Lagerstroemia minuticarpa in the Indian Eastern Himalaya. Journal of Environmental Management. 2019; 342: 299–307. 10.1016/j.jenvman.2019.05.036 31102897

[16] Brown G, Brabyn L. The extrapolation of social landscape values to a national level in New Zealand using landscape character classification. Applied Geography. 2012; 35(1–2): 84–94. 10.1016/j.apgeog.2012.06.002

[17] Song YF, Lu YJ, Liu TJ, Li HP, Yue ZW, Liu HW, et al. Variation of vegetation fractional coverage and its relationship with climate in a desert steppe: optimization of farmland layout in a farming–pastoral ecotone using the ecological suitability index. Ecological Engineering. 2020; 150: 105834. 10.1016/j.ecoleng.2020.105834

[18] Yan SJ, Wang X, Cai YP, Li CH, Yan R, Cui GN, et al. An Integrated Investigation of Spatiotemporal Habitat Quality Dynamics and Driving Forces in the Upper Basin of Miyun Reservoir, North China. Sustainability. 2018; 10(12): 4625–4641. 10.3390/su10124625

[19] Leinster T. Cobbold CA. Measuring diversity: the importance of species similarity. Ecology. 2012; 93(3): 477–489. 10.1890/10-2402.1 22624203

[20] Vallecillo S, Maes J, Polce C, Lavalle C. A habitat quality indicator for common birds in Europe based on species distribution models. Ecological Indicators. 2016; 69: 488–499. 10.1016/j.ecolind.2016.05.008

[21] Horne BV. Density as a misleading indicator of habitat quality. Journal of Wildlife Management. 1983; 47(4): 893–901. 10.2307/3808148

[22] Kempton RA. The structure of species abundance and measurement of diversity. Biometrics. 1979; 35(1): 307–321. 10.2307/2529952

[23] Jost L. Partitioning diversity into independent alpha and beta components. Ecology. 2007; 88(10): 2427–2439. 10.1890/06-1736.1 18027744

[24] He JH, Huang JL, Li C. The evaluation for the impact of land use change on habitat quality: A joint contribution of cellular automata scenario simulation and habitat quality assessment model. Ecological Modelling. 2017; 366: 58–67. 10.1016/j.ecolmodel.2017.10.001

[25] Li FX, Wang LY, Chen ZJ, Clarke KC, Li MC, Jiang PH. Extending the SLEUTH model to integrate habitat quality into urban growth simulation. Journal of Environmental Management. 2018; 217: 486–498. 10.1016/j.jenvman.2018.03.109 29631238

[26] Sharp R, Tallis HT, Ricketts T, Guerry AD, Wood SA, Chaplin-Kramer R, et al. InVEST 3.7.0.post27+ug.h0fb6c74c6697 User’s Guide. The Natural Capital Project, Stanford University, University of Minnesota, The Nature Conservancy, and World Wildlife Fund. 2018.

[27] Chu L, Sun TC, Wang TW, Li ZX, Cai CF. Evolution and Prediction of Landscape Pattern and Habitat Quality Based on CA-Markov and InVEST Model in Hubei Section of Three Gorges Reservoir Area (TGRA). Sustainability. 2018; 10(11): 3854–3881. 10.3390/su10113854

[28] Firozjaei MK, Sedighi A, Argany M, Jelokhani-Niaraki M, Arsanjani JJ. A geographical direction-based approach for capturing the local variation of urban expansion in the application of CA-Markov model. Cities. 2019; 93: 120–135. 10.1016/j.cities.2019.05.001

[29] Palmate SS, Pandey A, Mishra SK. Modelling spatiotemporal land dynamics for a trans-boundary river basin using integrated Cellular Automata and Markov Chain approach. Applied Geography. 2017; 82: 11–23. 10.1016/j.apgeog.2017.03.001

[30] Zhou L, Dang XW, Sun QK, Wang SH. Multi-scenario simulation of urban land change in Shanghai by random forest and CA-Markov model. Sustainable Cities and Society. 2020; 55: 102045. 10.1016/j.scs.2020.102045

[31] Cao YH, Xia YX, Mao GX, Cai AN, Liu CM. Research on regional development difference and collaborative development strategy of the Huaihe River Eco-economic Belt. Economic Geography. 2019; 39(9): 213–221.

[32] National Development and Reform Commission, 2018. Development plan of the Huai River Economic Belt.

[33] Xu XL. China GDP spatial distribution kilometer grid data set. Data Registration and publishing System of Resources and Environmental Sciences Data Center, Chinese Academy of Sciences, 2017.

[34] Xu XL. China population spatial distribution kilometer grid data set. Data Registration and publishing System of Resources and Environmental Sciences Data Center, Chinese Academy of Sciences, 2017.

[35] Mansour S, Al-Belushi M, Al-Awadhi T. Monitoring land use and land cover changes in the mountainous cities of Oman using GIS and CA-Markov modelling techniques. Land Use Policy. 2020; 91: 104414. 10.1016/j.landusepol.2019.104414

[36] Eastman JR. Idrisi selva tutorial, manual version 17. Idrisi Production, Clark Labs-ClarkUniversity, 2012.

[37] Tang F, Fu MC, Wang L, Zhang PT. Land-use change in Changli County, China: Predicting its spatio-temporal evolution in habitat quality. Ecological Indicators. 2020, 117: 106719.

[38] Moreira M, Fonseca C, Vergílio M, Calado H, Gil A. Spatial assessment of habitat conservation status in a Macaronesian island based on the InVEST model: a case study of Pico Island (Azores, Portugal). Land Use Policy. 2018; 78: 637–649. 10.1016/j.landusepol.2018.07.015

[39] Bai LM, Xiu CL, Feng XH, Liu DQ. Influence of urbanization on regional habitat quality: a case study of Changchun City. Habitat International. 2019; 93: 102042. 10.1016/j.habitatint.2019.102042

[40] Febbraro MD, Sallustio L, Vizzarri M, Rosa DD, Lisio LD, Loy A, et al. Expert-based and correlative models to map habitat quality: Which gives better support to conservation planning? Global Ecology and Conservation. 2018; 16: e00513. 10.1016/j.gecco.2018.e00513

[41] Han YW, Kang WM, Thorne J, Song Y. Modeling the effects of landscape patterns of current forests on the habitat quality of historical remnants in a highly urbanized area. Urban Forestry & Urban Greening. 2019; 41: 354–363. 10.1016/j.ufug.2019.04.015

[42] Wang N, Chen F, Yu B, Qin YC. Segmentation of large-scale remotely sensed images on a Spark Platform: a strategy for handling massive image tiles with the MapReduce model. ISPRS Journal of Photogrammetry and Remote Sensing. 2020; 162: 137–147. 10.1016/j.isprsjprs.2020.02.012

[43] Sallustio L, De Toni A, Strollo A, Di Febbraro M, Gissi E, Casella L, et al. Assessing habitat quality in relation to the spatial distribution of protected areas in Italy. Journal of Environmental Management. 2017; 201: 129–137. 10.1016/j.jenvman.2017.06.031 28651222

[44] Aneseyee AB, Noszczyk T, Soromessa T, Elias E. The InVEST habitat quality model associated with land use/cover changes: A qualitative case study of the WinikeWatershed in the Omo-Gibe Basin Southwest Ethiopia. Remote Sensing. 2020; 12(7): 1103. 10.3390/rs12071103

[45] Liu D, Gong QW, Yang WJ. The Evolution of Farmland Protection Policy and Optimization Path from 1978 to 2018. Chinese Rural Economy. 2018; 12: 37–51.

[46] Ren JL, Wang YP, Cheng Y. From ecological environmental protection to ecological civilization construction: A review and prospect. Journal of Shandong University(Philosophy and Social Sciences). 2018; 6: 27–39.

[47] Gao JX. How China will protect one-quarter of its land. Nature. 2019; 569: 457. 10.1038/d41586-019-01563-2 31114090

[48] Wang H, Li WW, Huang W, Nie K. A Multi-Objective Permanent Basic Farmland Delineation Model Based on Hybrid Particle Swarm Optimization. ISPRS International Journal of Geo-Information. 2020; 9(4): 243–267.

